# Assessing the Accuracy of Bed-Occupancy With a tina.care Bed Sensor in Hospital Wards and Home Care Settings: A Pilot Study

**DOI:** 10.1109/OJEMB.2025.3548838

**Published:** 2025-03-07

**Authors:** Tomáš Kulhánek, Kvetoslava Hošková, Jitka Feberová, Miroslav Malecha

**Affiliations:** Bonitoo s.r.o. 190 00 Czechia; First Faculty of MedicineCharles University 69729 128 53 Prague Czechia; Thomayer Faculty Hospital 140 59 Prague Czechia; First Faculty of MedicineCharles University 69729 140 59 Prague Czechia; Bonitoo s.r.o. 190 00 Prague Czechia

**Keywords:** mmwave sensor, contact-less monitoring, bed occupancy

## Abstract

*Goal:* This pilot study aims to assess accuracy in detecting patient presence or absence by using a bed sensor based on mmwave radar technology above the patient bed. *Methods:* Patients and healthy volunteers were observed during their presence or absence in a bed in hospital and home location. These observations were compared with data coming from bed sensor monitoring patient presence using tina.care bed sensor ASWA. *Results:* A total of 53 different observations were performed during the study period and the bed sensor reached accuracy of 94%, precision of 90%, sensitivity of 99% and specificity of 89% to detect presence or absence of patients in a bed. *Conclusions:* The sensor demonstrated strong performance in detecting patient presence in bed, with reasonable specificity and low false negatives. Further research should assess bed-exit and bed-entry events, system's accuracy in a larger cohort, its impact on patient care, and the precision of vital health parameters measured by the sensor in order to compare it with similar studies.

## I. Introduction

The he aging population in developed countries presents substantial challenges for healthcare systems, requiring innovative solutions to meet increasing care demands. National and international studies highlight several key issues, many of which can be addressed through the adoption of innovative technologies. One of the identified approaches is to meet the growing demand for institutional care by using technology to monitor the condition and activity of patients in bed, whether in hospital or home-care settings. [1], [15], [16].

The purpose of implementing such a monitoring system is to streamline and enhance the efficiency of healthcare staff by providing them with real-time information about the patient's condition, enabling more informed decisions about when and how to intervene. Various technologies are currently used to monitor whether a patient is in bed. Wearable devices [2], [3], [4], [6] are attached to the patient's body and can trigger an alarm if the patient moves outside a designated area. Pressure sensors [5], [6], [9] use e.g., some sort of inflated mat to detect pressure changes under selected areas such as a bed mattress or floor. It can trigger alarms and, in some cases, estimate vital signs from these pressure changes. Radar sensors [6], [10] transmit and receive radar waveforms, estimating the presence, distance, and movement of objects within their sensing range. These sensors can detect micro-movements and estimate vital signs. Additionally, recent advancements in integrating radar sensors with machine learning techniques have enabled the reconstruction of 3D point clouds representing a scene, which can be used to recognize patient activity, gait, falls, and posture within the radar's range [17], [18].

Infrared sensors detect the infrared radiation emitted by the human body, identifying presence and motion patterns [9], [10]. The most informative but also intrusive and non-private are camera sensors [6], [11], [14], which use camera images to detect and estimate patient position and movement.

A key motivation behind these non-invasive monitoring systems are:

• to keep control of the patient's state and behavior in a hospital ward, i.e., his/her compliance with recommended regime and its presence in the bed
• the prevention of bed falls by early detection of a patient's attempts to exit the bed and notification of health care staff that may intervene.

The previously mentioned studies focus mainly on bed-exit or bed-entry events. However, to the best of our knowledge, no study has specifically assessed the accuracy and precision of bed-occupancy detection using simple radar sensors in hospital or home-care settings. In other words, if a patient remains in bed continuously for two hours, how accurately does a sensor detect this continuous presence or absence?

Therefore, in this study, we conduct a pilot accuracy evaluation of millimeter-wave (mmWave) radar sensors, which operate at high frequencies, to detect the presence of a patient in bed for observed periods of up to one hour. Additionally, we assess transition events, such as bed-entry and bed-exit, as these have been evaluated in several studies addressing fall risk. When placed above a bed, this sensor can determine whether a person is present and provide additional information to healthcare staff. The primary goal is to detect patient absconding, evaluate bed-exit events, and assess other unusual behaviors, which are common concerns for elderly, disoriented, or otherwise vulnerable patients. The secondary goal is to obtain performance data for comparison with future studies using other advanced sensors and technologies.

The following sections describe the methods used in the study, the pilot participant cohort, and the performance metrics for monitoring patient presence in bed, as well as assessing the detection of transition events such as bed-entry and bed-exit.

## II. Materials and Methods

The tina.care bed sensor ASWA was used in this study. It consists of an mmWave radar sensor that sends high-frequency signals and, based on the Doppler effect, estimates an object's distance and relative speed. The data are numerically encoded as several quantities, which are then transmitted via a Wi-Fi network to a time-series database approximately six times per minute or more. The data are subsequently aggregated and reported to healthcare staff or home caregivers, linked only to the identification of the sensor-equipped bed. No other data are stored or reported, and no personal or identifiable information about the patient in the bed is known to the system. We have selected a specific quantity and threshold to determine the presence or absence of a patient in bed.

**Fig. 1.**
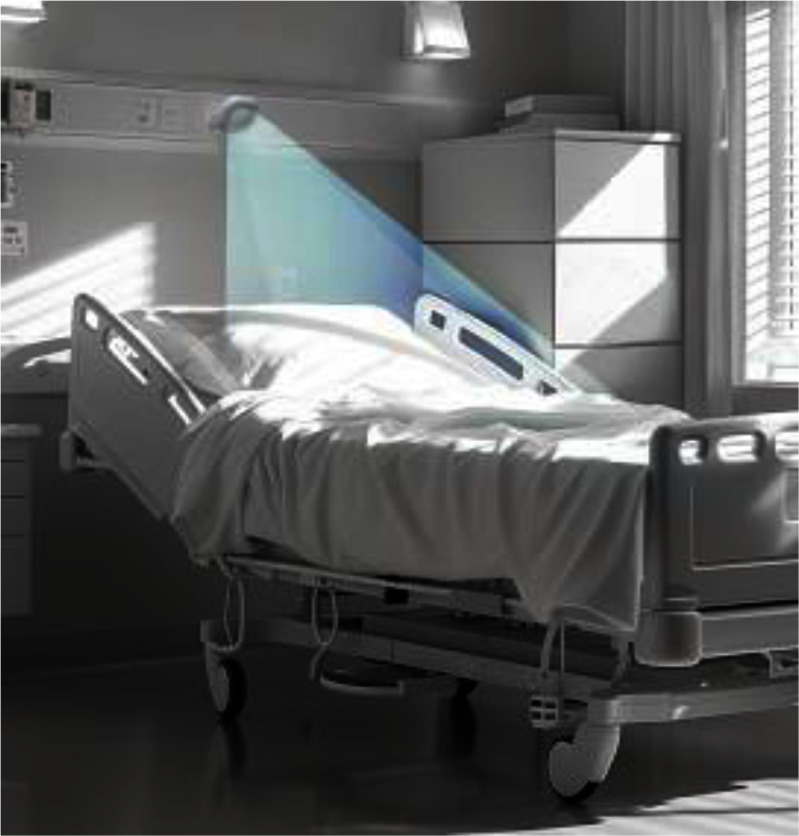
Recommended placement of the sensor is from 1 to 1.3 meters above the bed and 0 to 0.4 meters behind the head of the bed, and angled at 45° toward the bed surface. The sensor range is visualized as a cone of blue light.

All the sensors are placed from 1 to 1.3 meters above the bed and 0 to 0.4 meters behind the head of the bed, e.g., on the wall as in Fig. 1. Small number of sensors were positioned from the side of the bed on the wall rather from the head, however, this installation did not result in significantly different measurements thus were not excluded from the study.

The sensor is an IoT device that reports every 10 s several sensor measurements to a time series database. We selected one of the sensor values to determine the presence or absence of a patient in the bed.

A total of 32 volunteer patients and/or healthy volunteers were monitored, with a total of 53 observations. The patients were from the geriatric and follow-up care ward of the university hospital, where the pilot deployment of the sensor took place. All patients provided written consent acknowledging that the hospital is a teaching institution where educational and research activities may occur during their stay, and they retained the right to verbally decline participation in any research or educational activity at any time during their treatment. Only patients who additionally gave verbal approval to be observed for this particular pilot study were included. Selection was based solely on the patients' ability to provide consent.

Healthy volunteers included one elderly person in a home-care setting and three healthy adults. There were no significant differences in the installation of sensors in home-care settings compared to hospital wards. Participants did not follow a specific protocol; only general observations of activity in and around the bed were logged.

The data recorded and stored during the study do not contain any personal or sensitive information; they are fully anonymous and include only timestamps, bed identification, and the selected quantity used to determine presence or absence in bed. If more specific information about a participant was needed, access was restricted to healthcare staff or home caregivers. No such information pairing was conducted during this pilot study.

**Fig. 2.**
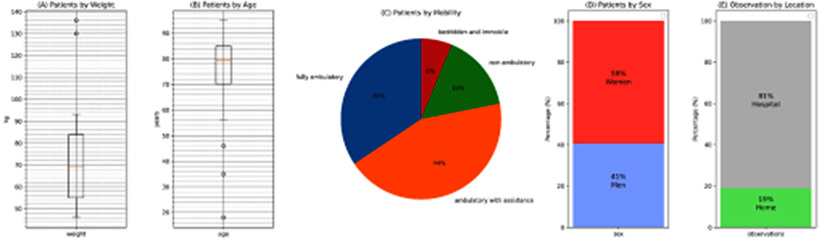
(a) Distribution of patients by weight in the study with mean value of 70 kg, two patients were obese with weight over 130 kg (b) Distribution of patients by age with mean age of 80 year with youngest 16 year old and oldest 96 years old. (c) Distribution of patient based on their mobility with majority of 44% ambulatory with assistance, (d) Distribution of patients by sex in the study with 59% women and 41% men, and (e) Distribution of 53 observations by location, 81% hospital observations and 19% home care observations.

All patients and volunteers were of white European descent, primarily from Czechia and Slovakia. The composition and basic information of the testing cohort is in Fig. 2.

To compare sensor measurements with ground truth, the following "gold standard" methods were considered: (1) camera recordings, (2) pressure sensors, and (3) observation by a human observer. Camera recordings and pressure sensors were not permitted in the hospital ward; therefore, only observations by healthcare staff or home caregivers were allowed in this pilot study. Thus, each observation took from 20 up to 60 minutes and assessed the presence or absence of a person in a bed, position in a bed and whether the 2nd person was near bed, like a nurse, staff member, or visitor near the bed. These observations were used to generate 10-second intervals indicating whether the patient was in or out of bed during each interval. The logs from each observation were exported into a standardized structured file in JSON format containing time, position of patient and device which the log refers to.

We consider these time-stamped logs as the ground truth for comparison with the corresponding sensor values stored in the time series database to determine true positives (TP), false positives (FP), true negatives (TN), and false negatives (FN). The following performance metrics including Positive Likelihood Ratio (LR+) and Negative Likelihood Ratio (LR-) were computed using these equations:

\begin{align*}
&{\mathrm{Sensitivity = TP/}}\left( {{\mathrm{TP + FN}}} \right) \tag{1}\\
&{\mathrm{Specificity = TN/}}\left( {{\mathrm{TN + FP}}} \right) \tag{2}\\
&{\mathrm{Accuracy = }}\left( {{\mathrm{TP + TN}}} \right){\mathrm{/}}\left( {{\mathrm{TP + TN + FP + FN}}} \right) \tag{3}\\
&\text{Precision}\left( {{\mathrm{Positive Predictive Value}}} \right){\mathrm{ = TP/}}\left( {{\mathrm{TP + FP}}} \right) \tag{4}\\
&{\mathrm{Negative Predictive Value = TN/}}\left( {{\mathrm{TN + FN}}} \right) \tag{5}\\
&{\mathrm{Prevalence = }}\left( {{\mathrm{TP + FN}}} \right){\mathrm{/size\_of\_study}} \tag{6}\\
&{\mathrm{Positive Likelihood Ratio}}\left( {{\mathrm{LR + }}} \right)\\
&\quad{\mathrm{ = Sensitivity/}}\left( {{\mathrm{1 - Specificity}}} \right) \tag{7}\\
&{\mathrm{Negative Likelihood Ratio}}\left( {{\mathrm{LR - }}} \right)\\
&\quad{\mathrm{ = }}\left( {{\mathrm{1 - Sensitivity}}} \right){\mathrm{/Specificity}} \tag{8}\\
&{\mathrm{F1 Score = 2x}}\left( {{\mathrm{Precision x Sensitivity}}} \right)\\
&\quad{\mathrm{/}}\left( {{\mathrm{Precision + Sensitivity}}} \right) \tag{9}
\end{align*}

## III. Results

Patients and volunteers did not follow a specific protocol but engaged in their usual activities, such as sleeping, reading in bed, leaving the bed for rehabilitation or other purposes, and returning to bed.

Additionally, we excluded those log entries where the patient was out of bed and a second person, such as a staff member, was near the bed (e.g., making the bed). This was done to avoid potential false negatives, as these scenarios do not reflect the intended use case of monitoring the patient's presence when no staff is present in the room. We also excluded logs when the patient was sitting on a bed. This is because bed sitting is currently evaluated on higher levels of the system using a combination of sensor values which is not the primary focus of this particular study.

We compared the remaining observation logs with the sensor measurements and obtained true positive (TP), false positive (FP), true negative (TN), and false negative (FN) values, as presented in the confusion matrix in Table I for bed occupancy.

**TABLE I:** Bed Occupancy Observation vs Sensor

Sensor/Human observation	In Bed	Out of Bed	Total
Sensor in bed	3642 (TP)	386 (FP)	4028
Sensor out bed	37 (FN)	3220 (TN)	3257
Total	3679	3606	

Using the values above, the performance metrics for bed occupancy were calculated according to equations (1) through (9), as shown in Table II.

**TABLE II:** Performance Metrics of the tina.care Bed Sensor ASWA for Determining Whether a Patient Is in or Out of Bed

metric	value
Sensitivity	99 %
Specificity	89 %
Accuracy	94 %
Precision	90 %
Negative Predictive Value	99 %
Prevalence	38 %
LR+	9.25
LR-	0.01
F1 Score	95 %

In this study, prevalence refers to the proportion of time the patient was present in bed during observations. Thus 38% of the observed time the patients were in bed. Sensitivity of 99% means that the bed sensor was able to correctly detect the presence of a patient in bed 99% of the time.

**Fig. 3.**
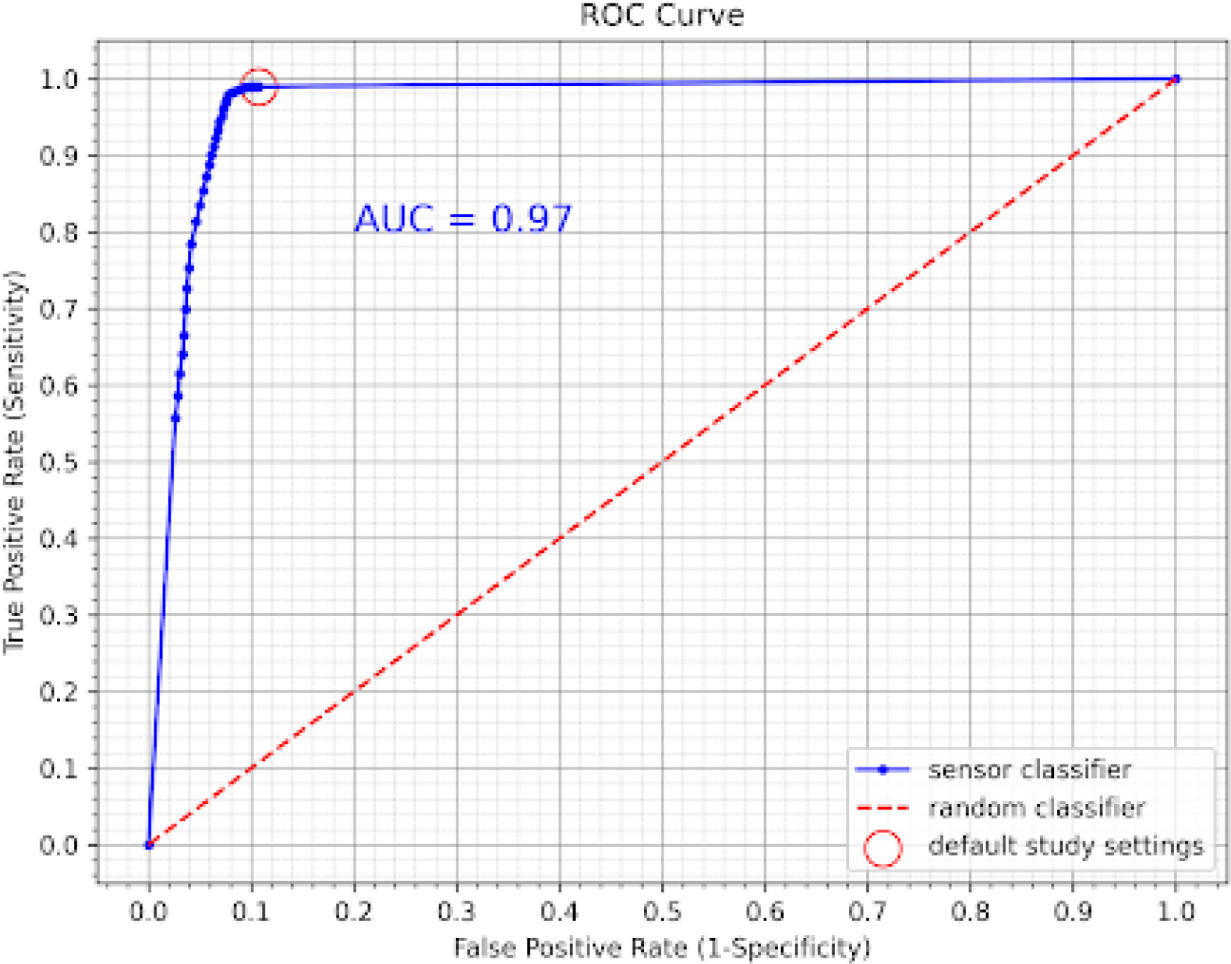
Receiver operating characteristic (ROC) analysis and curve for sensor classifiers based on varying sensor threshold values. The minimal (default) threshold shows a reported sensitivity of 99% and specificity of 89%. As the threshold value increases, sensitivity drops to 56%, while specificity increases to 97%. The area under the curve (AUC) is estimated at 0.97.

Additionally, we retrospectively adjusted the threshold of the sensor value used to determine in-bed and out-of-bed status. We then recalculated the sensor values and compared them to the corresponding observations. This allowed us to obtain true positive rates (sensitivity) and false positive rates (1-specificity) based on the adjusted threshold. The receiver operating characteristic (ROC) curve, reflecting these threshold changes, is shown in Fig. 3.

While some observations were performed in a hospital environment and others in home care, we calculated the performance metrics also separately for each environment in order to have comparison between these two settings. The comparison is shown in Tables III and IV.

**TABLE III:** Bed Occupancy Observation vs Sensor in Hospital and Home Care Settings Separately

Sensor/Human observation	In Bed Hospital	Out Bed Hospital	Total	In Bed Home	Out Bed Home	Total Home
Sensor in bed	2153 (TP)	333 (FP)	2486	1489 (TP)	53 (FP)	1542
Sensor out bed	13 (FN)	2003 (TN)	2016	24 (FN)	1217 (TN)	1241
Total	2166	2336		1513	1270

**TABLE IV:** Performance Metrics of the tina.care Bed Sensor ASWA for Determining Whether a Patient Is in or Out of Bed for Hospital and Home-Care Settings

Metric	Value for hospital observations	Value for home-care observations
Sensitivity	99 %	98 %
Specificity	86 %	96 %
Accuracy	92 %	97 %
Precision	87 %	97 %
Negative Predictive Value	99 %	98 %
Prevalence	32 %	53 %
LR+	6.97	23.58
LR-	0.01	0.02
F1 Score	93 %	97 %

## IV. Discussion

Overall, the system performed well in detecting patient presence in bed. The ROC curve indicates that there is a threshold at which accuracy could slightly improve if sensitivity and specificity are given equal weight. The relatively lower specificity is partly due to the sensor's settings, which include an intentional delay between the patient leaving the bed and the sensor triggering the ‘out of bed’ event to prevent false alarms. In this case, sensitivity was prioritized over specificity to minimize false negatives, where the sensor incorrectly triggers an ‘out of bed’ alert even though the patient is still in bed. This approach also addresses alarm fatigue, a concern highlighted in other studies.

The 81% of observations were performed in pilot deployment in a hospital environment, where additional challenges are present. When comparing performance metrics between hospital and home-care environments, slightly better results were observed in home-care settings. This improvement was due to the fixed positioning of the sensor above the bed and the more consistent behavior of patients and volunteers in the home environment. In hospitals, the sensors were mounted on walls near mobile beds, and during bed or patient manipulation, the sensors occasionally ended up in suboptimal positions.

A similar study assessed performance metrics for transition events, bed-exit and bed-entry using a wearable sensor WISP [3]. We tried to assess bed-exit and bed-entry transitions from the performed observation in order to compare such statistics, however, due to the low number of observed bed-exit and bed-entry events, the collected data were insufficient to ensure statistical robustness. As a result, the performance metrics for these events are not reported, as they do not meet the criteria for statistical significance. The observations were passive, and patients and volunteers were not instructed to follow a specific protocol for entering or exiting their bed. Further observations on a larger cohort of patients and volunteers are needed to accurately assess the radar sensor's ability to detect transition events.

During consultations with nurses and other healthcare staff, it was emphasized that alerting for unusual patient behavior, tailored to the patient's mobility level, is crucial. For example, in immobile or non-ambulatory patients, even a brief absence from bed may indicate a serious issue, such as a fall. However, for fully ambulatory patients or those with assistance, a short absence from bed is generally not concerning.

In summary, radar sensors are particularly well-suited for continuously monitoring patient presence and providing valuable insights into vital health parameters and ward bed occupancy.

The sensor used in the study may be used to estimate vital health parameters of patients contactless [7], [8] as the similar studies report precision of 95% for breath and 91% for heart rate of a lying down person [12]. Further studies may be required to determine the precision of vital health parameters obtained from radar sensors, particularly in patients commonly found in geriatric wards or home-care settings. Further studies may also assess the system's accuracy in a larger cohort, evaluate its impact on patient care quality, and assess transition events like bed-exit and bed-entry in order to compare with other technologies used or tested in the healthcare environment.

## V. Conclusion

The majority of similar studies on different sensors primarily focus on bed-exit or bed-entry events. However, the accuracy of long-term patient presence or absence in bed has rarely been evaluated. Within this pilot study, the simple mmWave sensor demonstrated strong performance in detecting patient presence in bed, with reasonable specificity and a low false-negative rate.

Further research on a larger cohort of patients is needed to assess bed occupancy more comprehensively, including bed-exit and bed-entry events, their impact on patient care, and the accuracy of vital health parameter estimation. This study may serve as a valuable benchmark for future comparisons with similar studies using different sensors or technologies, such as sensors with improved body position detection, AI and machine learning-based analysis, and 3D point cloud reconstruction for better estimate of patient state.

## Supplementary Materials

The supplementary materials including raw data from observation and Python scripts to perform analysis and statistical computation are available on GitHub and archived in Zenodo [19].

## Ethics Statement

This work involved human subjects. All ethical and experimental procedures and protocols were approved by the Thomayer University Hospital. Patients provided written consent acknowledging that the hospital is a teaching institution where educational and research activities may take place during their stay. However, patients retained the right to verbally decline participation in any educational and research activity at any time during their treatment.

## Author Contribution

T.K. designed the study, carried out the experimental observations, and analyzed and interpreted the results. K.H. supervised the experiments and observations. J.F. contributed to the statistical analysis and reviewed the methods. M.M. conceived the study and contributed to the design and setup of the experiments. T.K. wrote the initial draft of the manuscript, and all authors read and approved the final version.

## Conflict of Interest

This work was supported by Bonitoo s.r.o, the maker of the tina.care bed sensor ASWA. T.K. and M.M. disclose their affiliation with Bonitoo s.r.o during the study. Bonitoo s.r.o is the maker of the tina.care bed sensor ASWA. The remaining authors K.H. and J.F. declare that the research was conducted in the absence of any commercial or financial relationships that could be construed as a potential conflict of interest.
